# P-471. Clinical and Laboratory Characteristics of the Stockholm HTLV Cohort

**DOI:** 10.1093/ofid/ofae631.670

**Published:** 2025-01-29

**Authors:** Anna-Karin Svensson, Jan Vesterbacka, Sara Gredmark-Russ, Alexandros Petropoulos, Anna Nordlander, Piotr Nowak

**Affiliations:** Karolinska universityHospital infectious disease unit, Stockholm, Stockholms Lan, Sweden; Karolinska universityHospital infectious disease unit, Stockholm, Stockholms Lan, Sweden; Karolinska universityHospital infectious disease unit, Stockholm, Stockholms Lan, Sweden; Karolinska university Hospital infectious disease unit, stockholm, Stockholms Lan, Sweden; Karolinska university Hospital infectious disease unit, stockholm, Stockholms Lan, Sweden; Karolinska universityHospital infectious disease unit, Stockholm, Stockholms Lan, Sweden

## Abstract

**Background:**

The prevalence of HTLV infection in Sweden is presumed to be low, however the impact of demographic changes during the last 20 years on HTLV prevalence is unknown. In our study we describe clinical and laboratory characteristics of patients with HTLV -infection followed at our outpatient clinic at Karolinska University Hospital.

**Methods:**

The clinical and laboratory data has been extracted from medical records of patient with HTLV infection between 2000-2024. We established a database which included demographics, mode of transmission, proviral HTLV load (PVL), occurrence of HTLV-1 associated myelopathy/tropical spastic paraparesis and Adult T cell leukemia (HAM/TSP and ATL), comorbidities and immunological and routine laboratory markers.

**Results:**

We identified 101 patients with HTLV infection followed at our site (80% HTLV-1, 20% HTLV-2). Fifty-tree percent (n=54) were women. The average age in the cohort was 53,6 years (23-81). Twenty-nine percent (n=29) of the patients were born in Sweden, while the rest had other countries of origin (35% Middle east; 17% South America; 11% Africa ). HTLV infection was predominantly diagnosed through contact testing (34%), blood donor screening (32%), and pre-in vitro fertilization screening (32%), while clinical symptom investigation accounted for 12% of cases. Eleven individuals were HIV positive and predominantly co infected with HTLV-2. Of note, within the subset of patients born in Sweden, we identified four cases of mother-to-child transmission (MTCT) of HTLV.

Until January 2024 ten individuals (10%) were diagnosed with HAM/TSP and two individuals with ATL. Patients with HAM/TSP had a higher average PVL as compared to the rest of the cohort (20,9% vs 7,8%).

Furthermore, we conducted a quantitative analysis of regulatory T-cells in 26 HTLV-1 infected patients in peripheral blood using flow cytometry. Fifteen patients (58%) had elevated T-reg percentage (ranging from 12% to 51%) and higher PVL. Notably all three HAM/TSP patients were in the elevated T reg group.

**Conclusion:**

In our cohort, we report a high percentage of patients with HAM/TSP and ATL. Notably, we found four cases of MTCT in patients born in Sweden. We also found a higher abundance of regulatory T-cells in the patient’s group with higher PVL and more prevalent occurrence of symptoms.

**Disclosures:**

**All Authors**: No reported disclosures

